# Seasonal and Annual Change in Physiological Ocular Growth of 7- to 11-Year-Old Norwegian Children

**DOI:** 10.1167/iovs.64.15.10

**Published:** 2023-12-08

**Authors:** Nickolai G. Nilsen, Stuart J. Gilson, Helene Lindgren, Marianne Kjærland, Hilde R. Pedersen, Rigmor C. Baraas

**Affiliations:** 1 National Centre for Optics, Vision and Eye Care, Faculty of Health and Social Sciences, University of South-Eastern Norway, Kongsberg, Norway

**Keywords:** seasonal changes, physiological eye growth, refractive error development, myopia, choroidal thickness

## Abstract

**Purpose:**

To investigate seasonal and annual change in physiological eye growth in Norwegian school children.

**Methods:**

Measurements of ocular biometry, non-cycloplegic spherical equivalent autorefraction (SER), and choroidal thickness (ChT) were obtained for 92 children (44 females) aged 7 to 11 years at four time points over a year (November 2019–November 2020). Seasons (3- and 5-month intervals) were classified as winter (November–January), winter–spring (January–June), and summer–autumn (June–November). Cycloplegic SER was obtained in January and used to group children. The seasonal and annual changes were tested with a linear mixed-effects model (*P* values were adjusted for multiple comparisons).

**Results:**

All the children experienced annual ocular growth, irrespective of SER, but less so during the summer–autumn. The baseline SER was lower (*P* < 0.001), axial length (AL) was longer (*P* < 0.038), and choroids were thicker in 10- to 11-year-old than 7- to 8-year-old mild hyperopes (*P* = 0.002). Assuming mild hyperopes (*n* = 65) experience only physiological eye growth, modeling revealed seasonal and annual increases in AL across sex and age (*P* < 0.018), with less change during the summer–autumn than winter–spring. The 7- to 8-year-olds had a larger decrease annually and over winter–spring in SER (*P* ≤ 0.036) and in ChT over winter–spring than the 10- to 11-year-olds (*P* = 0.006).

**Conclusions:**

There were significant seasonal and annual changes in AL in children who had physiological eye growth irrespective of age within this cohort. Annual changes in SER and seasonal choroidal thinning were only observed in 7- to 8-year-old children. This indicates continued emmetropization in 7- to 8-year-olds and a transition to maintaining emmetropia in 10- to 11-year-olds.

There is growing evidence supporting the theory that time spent outdoors and increased daylight exposure could be major factors for normal emmetropization during childhood and for maintaining emmetropia throughout adolescence and, consequently, delaying or preventing myopia onset.^1^^,^^2^ Supporting evidence has been provided by cluster randomized trials whereby compulsory outdoor time during recess at school has been tested as the intervention and shown to be successful at decreasing the incidence of myopia.^3^^–^^5^ Objective measures of light exposure during one of these cluster randomized trials reported a strong association between the protective effect of outdoor time and the duration and intensity of the light,^4^ akin to data from animal models of myopia such as rhesus monkeys.^6^ Furthermore, when compared with Southeast Asia, considerably lower myopia prevalence has been reported in Scandinavia (<13% in 16- to 19-year-olds, 10% in 12-year-olds),^7^^,^^8^ where compulsory outdoor time during recess is the norm (irrespective of time of year).^7^^–^^9^ Data on adolescents and young adults from southeast Norway (latitude 60°N), where there are ≈12 hours more daylight available in summer than in winter,^10^ suggest that the delayed onset and low prevalence of myopia could be a result of children's eyes being adapted to seasonal variations in daylight availability.^7^^,^^11^ There is value in such a suggestion, as there are several reports on seasonal variation in myopia development with progression being slower during summer compared with winter,^12^^–^^16^ which has been linked to increased availability of daylight in summer rather than fewer school hours,^16^ or a combination of both.^15^

Animal studies have consistently shown that choroidal thickness may act as a biomarker of eye growth (for a review, see Troilo et al.^17^). Choroidal thickness has been shown to be affected by light exposure in both animals^18^^,^^19^ and human adults.^20^^–^^23^ The reported associations between less thickening or thinning of the choroid, increased axial length, and myopia in human studies of children aged 6 to 18 years^24^^–^^26^ imply that the choroid may act also as an eye growth biomarker in humans.^27^ Human emmetropization is reported to be influenced by visual experience and, in general, to be completed by 6 years of age,^28^ but it is not known if physiological ocular growth follows a similar seasonal pattern as that observed for myopic ocular growth. Physiological ocular growth is defined here as a two-phase process: normal eye growth as experienced by children who successfully emmetropize and, in the second phase, maintenance of emmetropia/mild hyperopia through coordinated growth (i.e., the eye grows in length while its crystalline lens flattens, thins, and loses optical power, but the refractive error remains unchanged).^28^^–^^31^ An 18-month-longitudinal study reported an inverse relationship between daylight exposure and change in axial length in 10- to 15-year-old children (*n* = 60 non-myopes)^32^ and a potential (but nonsignificant) seasonal variation with larger axial length (AL) changes and less thickening of the choroid in winter.^24^ The difference in daylight availability between seasons at the study location (Australia, latitude 27°S) was 3 hours. Taken together, it is reasonable to hypothesize that physiological eye growth follows seasonal variation in availability of daylight, if guided by the same mechanism as that observed for myopic ocular growth,^12^^–^^16^^,^^28^ with larger changes in axial elongation in winter than in summer. Determining to what degree physiological ocular growth and choroidal thickness follow a similar seasonal pattern as that observed for myopic ocular growth is required to better understand what differs between success and failure to maintain emmetropia, with failure leading to myopia.^28^ The aim of this study was to investigate seasonal and annual changes in physiological eye growth and choroidal thickness in a cohort of healthy 7- to 11-year-old schoolchildren, who have mandatory outdoor time during recess every school day, irrespective of season, and who live at a location (Norway, latitude 60°N) where there are large differences in daylight availability between winter and summer. Another aim was to shed light on whether children who experienced only physiological ocular growth were undergoing emmetropization or if they had transitioned to maintenance of emmetropia, whereby the refractive error remained unchanged (maintaining emmetropia/mild refractive error).

## Methods

### Participants

Ninety-two children (44 female; aged 7–11 years), who attended second and fifth grade (7–8 years old and 10–11 years old, respectively) at one primary school in Kongsberg, Norway, were enrolled in this 12-month prospective longitudinal study. The study was approved by the Regional Committee for Medical and Health Research Ethics (Southern Norway Regional Health Authority), and both parents/caregivers provided written consent for their child to participate. The study was carried out in accordance with the tenets of the Declaration of Helsinki. All children included in the study were healthy with no history of ocular disease as reported by their parents. Habitual distance high-contrast visual acuity was in the range −0.18 to 0.70 logMAR (TestChart 2000; Thomson Software Solutions, London, UK) and stereo acuity 15 to 480 seconds of arc (TNO Stereotest; Laméris Ootech, WC Ede, Netherlands). Three myopes (cycloplegic spherical equivalent autorefraction [SER] −1.25 to −0.50 D) and 11 significant hyperopes (cycloplegic SER +2.00 to <+3.00) were uncorrected and referred to the university eye clinic. None of the children who wore prescription correction had received any other optical treatment than single-vision spectacle correction.

### Outdoor Time Before and During the COVID-19 Lockdown

The children's weekdays start and end in the before- and after-school (BAS) program that is offered from 07:00 to 17:00. Most children (>63% in this municipality in 2020) attend this program,^33^ as primary caregivers are typically in full-time employment (85%).^34^ The 7- to 8-year-olds have structured teaching from 08:30 to 13:00 and the 10- to 11-year-olds from 08:30 to 13:45. All have a 15-minute recess in the morning and 30 minutes after lunch, and the older children have an additional 10-minute recess in the afternoon. The children must go outdoors during recess, irrespective of weather or time of year. It is reasonable to assume that most of the children will get 1 to 2 hours of outdoor time every day, even in midwinter (when combining outdoor time during recess and the BAS program), and Table 1 shows that this outdoor time coincides with daylight hours.

**Table 1.** Timing and Number of Months Between Study Visits, Range of Daylight Availability^10^ in Hours and Minutes Between Study Visit, Number of School Days and Number of Weekdays With Available Daylight in the Morning When the Children Are Walking to School, and From School Ending Until the Evening Right Up to Typical Bedtime

					# Weekdays With Daylight	
Season	# Months	Range of Daylight [Hours:Minutes]	# Nonschool Days^*^	# School Days	When Walking to School	Until Bedtime 20:30^†^
A1–W Autumn–Winter	2	07:25–06:40	24	25	0	0
W–S Winter–Spring	5	06:44–18:35	64	91	66	41
S–A2 Summer–Autumn	5	18:38–08:08	87	74	45	33
A1–A2 Annual	12		175	190	111	74

The study visits were in November 2019 (A1), January 2020 (W), June 2020 (S). and November 2020 (A2).

* Number of nonschool days includes weekends and holidays

† Recommended bedtime for Norwegian children aged 7 to 11 years of age.^87^

The COVID-19 lockdown lasted 6 weeks (March 12–April 20, 2020) for the second graders and 9 weeks (March 12–May 11, 2020) for the fifth graders.^35^^–^^37^ The rector of the school reported that homeschooling was scheduled as normal schooldays, including outdoor time during recess, but with no BAS program. Each child had their own tablet for participating in online learning, for doing and reporting on their school- and homework. In 2020, 96% of the Norwegian population and 99% of those aged 9 to 79 years had their own smartphone.^38^ Norwegian children in the relevant age group reportedly spent close to 4 hours per day online in 2020 (including school activity).^39^

### Data-Gathering Protocol

Repeated measures of body height, retinal imaging, ocular biometry, and autorefraction were obtained at baseline in November 2019 (autumn, A1), with follow-up measures obtained in January 2020 (winter, W), June 2020 (spring/summer, S), and the subsequent autumn, November 2020 (A2). Details about number of schooldays and availability of daylight are given in Table 1. The child's height was measured first (without footwear); thereafter, measurements of non-cycloplegic autorefraction (Nvision-K 5001 open-view autorefractor; Shin-Nippon, Tokyo, Japan) at a distance of 600 cm, followed by ocular biometry to measure corneal radius (CR) and AL (IOLMaster 700; Carl Zeiss Meditec AG, Jena, Germany); and lastly, optical coherence tomography (OCT) of the choroid (Spectralis OCT2-EDI; Heidelberg Engineering, Heidelberg, Germany). All measurements were obtained at school.

To minimize the effect of diurnal variations on ocular parameters,^40^ the children were measured between 11:00 and 14:30. Instruments were placed at approximately the same locations in a classroom at the school at each study visit with curtains kept closed to maintain similar light levels (measured to be 170–190 lux and 20–50 lux at the headrest of the autorefractor and OCT, respectively) to keep any effect of differing light levels on choroidal thickness (ChT) to a minimum.^20^ After biometry measurements and just before OCT imaging, the children watched a movie on a TV for 15 minutes at a 6-m distance for “accommodation washout,” to minimize the accommodative effect on choroidal thickness from previous near-work.^41^ The light levels at the location where the children were seated to watch the movie varied between 90 and 110 lux depending on the brightness of the movie's scenes.

OCT images were not acquired in November 2020 (A2) due to COVID-19 restrictions at the school, preventing the necessary contact time per child. Cycloplegic autorefraction (Huvitz HRK-8000A; Huvitz Co. Ltd., Gyeonggi-do, Korea) was measured once, in January 2020, 2 weeks after the January 2020 follow-up session, as this was the most suitable time for the school. Autorefraction was performed 30 minutes postinstillation of 1% cyclopentolate hydrochloride (Minims single dose; Bausch + Lomb, Bridgewater, NJ, USA). Children with lightly pigmented (blue to green) irides received one drop while those with more heavily pigmented irides received two drops.^42^ There were 92 children attending the baseline measures in November 2019; 91 of these attended the repeated measurement session in January 2020 (one child withdrew from the study), of whom 78 also attended on the day we obtained cycloplegic SER. Thirteen of the children were absent from school on the day cycloplegic measures were obtained. A total of 84 children completed the remaining repeated measurement sessions, with OCT images of sufficient quality for analysis obtained for 79 of these 84.

### OCT Measurement Protocol and Segmentation

**Figure 1.**
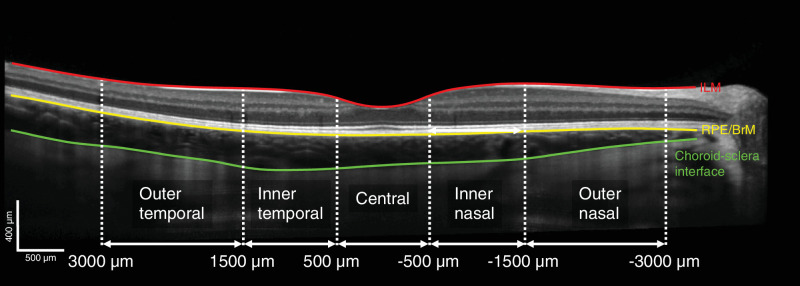
Example of segmentation of the retinal and choroidal thicknesses, which were defined as the area between the inner limiting membrane (ILM) and retinal pigment epithelium (RPE) layers, and the RPE layers and choroid–sclera interface, respectively. Mean thicknesses were extracted for the subfoveal (single A-scan), central (1-mm-wide), and the nasal and temporal inner (1-mm) and outer (1.5-mm) macular areas.

The OCT protocol included a six-line 30-degree radial scan centered at the fovea with 100 B-scans averaged at each orientation, with enhanced depth information. If there were fixation issues or a child could not sit still, the number of scans was reduced to either one (horizontal) or two (horizontal + vertical) line scans. The baseline measurement was set as a reference image for all subsequent measurements, using the instrument's retinal tracking system. For segmentation, a semiautomatic active contour method was fitted to the retinal and choroidal layers (as described previously^43^^,^^44^). Interrater reliability was assessed for the segmented subfoveal choroidal thickness (SFChT) by calculating the intraclass correlation (ICC) with a one-way model in R (irr package).^45^ ICC was 0.94 (*n* = 82; 95% confidence interval [CI], 0.91–0.96). Only the horizontal B-scans were used for analysis. The OCT scan's lateral scaling was corrected for each individual's ocular biometry (from an IOLMaster 700 measurement shortly before the OCT imaging) using a four-surface schematic eye model.^46^^,^^47^ The horizontal line scans were used to extract SFChT as well as mean ChT values for the central 1-mm area, the nasal and temporal inner 1-mm area (0.5–1.5 mm from the foveal center), and the outer 1.5-mm area (1.5–3 mm from the foveal center; Fig. 1). None of the children had any sign of ocular disease.

**Figure 2.**
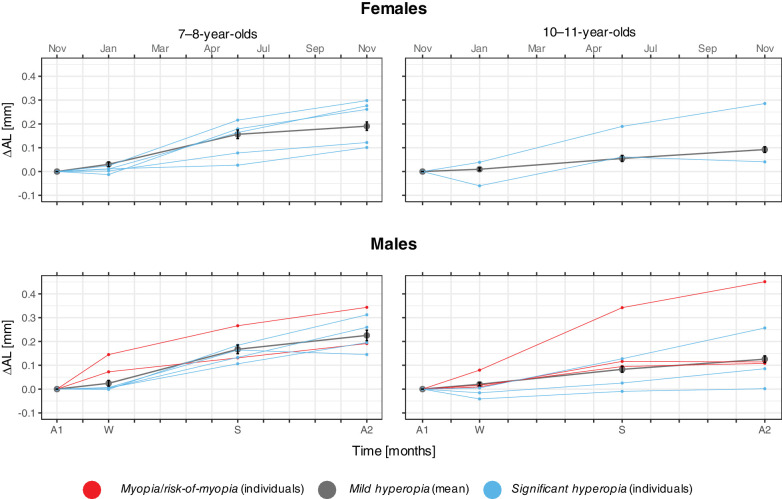
Change in axial length (∆AL) by sex and age groups across the measurement sessions: autumn 1 (A1), winter (W), summer (S), and autumn 2 (A2), normalized by the baseline value from A1. The *black lines* represent the average change for the *mild hyperopia* group (*n* = 30: 7- to 8-year-olds and *n* = 35: 10- to 11-year-olds), with the *error bars* representing the mean ± SE. Each *blue line* represents a child in the *significant hyperopia* group (*n* = 9: 7- to 8-year-olds and *n* = 5: 10- to 11-year-olds), while each *red line* represents a child in the *myopia/risk-of-myopia* group (*n* = 2: 7- to 8-year-olds and *n* = 3: 10- to 11-year-olds). There were no female children in either age group in the *myopia/risk-of-myopia* group.

### Data Analysis

Statistical analysis was performed using R statistical software, version 4.3.1.^48^ Parametric tests were used where the data had a normal distribution; otherwise, nonparametric tests were used. Statistical significance level was ɑ = 0.05. As there were no differences between OD and OS for measured values of ocular biometry (AL and CR) or non-cycloplegic and cycloplegic SER (*P* > 0.34), OD was arbitrarily chosen for analysis. CR was calculated as the average of two main meridians.

The children were classified into three groups: (1) *myopia or risk of myopia*, (2) *mild hyperopia*, and (3) *significant hyperopia*. The *myopia/risk-of-myopia* group was based on Zadnik's cutoff points for cycloplegic spherical refractive error: <+0.50 D for those aged 7 to 8 years and ≤+0.25 D for those aged 9 to 10 years.^49^ *Mild hyperopia* was defined as Zadnik's cutoff points above for each age group and ≤+2.00 D, as it was assumed that this group experienced physiological eye growth.^31^^,^^49^ *Significant hyperopia* was defined as >+2.00 D.

Linear mixed-effects models (LMMs, *lme4*^50^ and *lmerTest*^51^ R packages) were used to analyze the longitudinal data, using participant ID as a random effect and season, sex, and age groups as fixed effects, with a stepwise approach to assess significant predictors and interactions. A two-way ANOVA was used to examine differences between sex and age groups at baseline for a given ocular parameter or body height. Tukey's honestly significant difference (HSD) test was used to assess the specific significant differences between the groups. The *z*-score analysis with thresholds at ±1.96 (95% CI) were used to determine if the individuals in the *significant hyperopia* group or the *myopia/risk-of-myopia* group differed from those assumed to undergo physiological ocular growth (the *mild hyperopia* group) for changes in AL, SFChT, and body height.

LMM was used to estimate the within-session SD, and profiling the likelihood for the 95% CI, for cycloplegic (Huvitz HRK-8000A) and non-cycloplegic SER (Nvision-K 5001) and for axial length (IOLMaster 700), which were 0.07 D (0.065–0.073), 0.21 D (0.19–0.23), and 0.0048 mm (0.0046–0.0050, respectively; see Supplementary Table S1 for details). The values reported by HRK-8000A, Nvision-K 5001, and IOLMaster are based on the mean of five, five, and six single measurements, respectively.

## Results

### Baseline Characteristics

Table 2 shows the range of cycloplegic SER for the three SER groups by sex and age groups. Individuals were classified according to *cycloplegic* SER from winter (W), except for the 13 children who did not receive cycloplegic autorefraction, who were classified by a model that predicted cycloplegic SER from non-cycloplegic SER (*adjusted* SER; see Supplementary Material). The adjustment was made for these 13 and for all non-cycloplegic autorefraction measurements obtained at A1, S, and A2. Bland–Altman analysis shows that the mean difference between the *adjusted* and cycloplegic SER was −0.06D; Supplementary Fig. S1).

**Table 2.** Range of SER in Winter (*n* = 91) for Each of the Three SER Groups, Subgrouped by Sex and Age

	7- to 8-Year-Olds			10- to 11-Year-Olds		
Characteristic	*n*	Median	Range	*n*	Median	Range
*Significant hyperopia*	9	+2.26	+2.04–+3.49	6	+2.69	+2.01–+5.67
Female	5	+2.26	+2.14–+2.38	3	+2.65	+2.01–+5.18
Male	4	+2.51	+2.04–+3.49	3	+2.73	+2.44–+5.67
*Mild hyperopia*	31	+1.13	+0.59–+1.95	39	+0.84	+0.30–+1.82
Female	14	+1.00	+0.59–+1.95	21	+0.85	+0.30–+1.35
Male	17	+1.30	+0.65–+1.91	18	+0.80	+0.56–+1.82
*Myopia/risk* *of* *myopia*	3	−0.67	−0.98–+0.14	3	−0.20	−1.14–+0.20
Female	0	–	–	0	–	–
Male	3	−0.67	−0.98–+0.14	3	−0.20	−1.14–+0.20

SER grouping was according to cycloplegic autorefraction (*n* = 78) or by *adjusted SER* where cycloplegic autorefraction was unavailable (*n* = 13). There were no females in the *myopia/risk-of-myopia* group.

Table 3 shows baseline measures (A1) of AL, CR, and SFChT by sex- and age-per-SER group. There were no differences between sex groups in AL in the 7- to 8-year-old group, while the 10- to 11-year-old males had longer AL than females in both age groups (*F*(1, 66) = 4.58, *P* = 0.036, Tukey's HSD test *P* < 0.003). The females had, overall, a steeper CR than the males (*F*(1, 68) = 9.79, *P* = 0.003), with no differences between age groups. There were no differences in *adjusted* SER between females and males in the two age groups, but the larger AL in the 10- to 11-year-old group (Tukey's HSD test *P* = 0.038) corresponded with a significantly lower *adjusted* SER (difference of −0.58 D, *z* = 4.89, *r* = 0.52, *P* < 0.001). The choroid was significantly thicker in the subfoveal, central, and temporal (inner) areas compared to the nasal areas (inner and outer), with temporal outer and nasal inner areas being similar (*F*(5, 376) = 28.89, *P* < 0.001, Tukey's HSD test *P* < 0.003). Independent of area, the 10- to 11-year-olds had significantly thicker choroids than the 7- to 8-year-olds (difference of 24 µm, *F*(1, 376) = 10.14, *P* = 0.002), and females had significantly thicker choroids than males (difference of 21 µm, *F*(1, 376) = 8.70, *P* = 0.003). There was a weak but significant association between body height and AL at baseline (*R*^2^ = 0.09, *P* < 0.006), and the 10- to 11-year-olds were significantly taller than the 7- to 8-year-olds with no sex differences (ANOVA, *F*(1, 68) = 118, *P* < 0.001). There were no differences in ChT between areas, sex group, or age group. At baseline, there was no difference between the 84 who completed all four measurements and the 92 children who attended only the baseline session, which was not for *adjusted* SER, AL, CR (all *P* ≥ 0.869), or SFChT (*n* = 79, *P* ≥ 0.869).

**Table 3.** Baseline (A1) Measures of Body Height (*n* = 92), AL (*n* = 92), CR (*n* = 92), and SFChT (*n* = 84) per Age Group for Females and Males per Refractive Error Group

	7- to 8-Year-Olds					10- to 11-Year-Olds				
Characteristic	*n*	Mean	SD	Median	Range	*n*	Mean	SD	Median	Range
Height (cm)										
All	43	127.3	5.5	127.9	116.5–140.6	49	143.4	7.5	144.2	114.3–159.8
Female	19	127.1	5.0	125.3	121.0–137.0	25	143.6	6.6	144.4	129.4–159.8
Male	24	127.4	5.9	128.4	116.5–140.6	24	143.1	8.5	144.1	114.3–157.8
AL (mm)										
All	43	22.67	0.67	22.69	21.38–24.15	49	22.94	0.86	23.00	20.29–24.53
*Significant hyperopia*	9	22.13	0.44	22.11	21.38–22.75	6	22.07	1.10	22.43	20.29–23.23
Female	5	22.33	0.33	22.25	21.97–22.75	3	21.78	1.33	22.25	20.29–22.81
Male	4	21.87	0.47	21.81	21.38–22.48	3	22.36	1.02	22.62	21.24–23.23
*Mild hyperopia*	31	22.80	0.66	22.78	21.54–24.15	40	22.97	0.72	23.01	21.78–24.20
Female	14	22.65	0.47	22.80	21.54–23.21	22	22.57	0.52	22.54	21.78–23.71
Male	17	22.92	0.77	22.72	21.56–24.15	18	23.45	0.63	23.60	22.12–24.2
*Myopia/risk* *of* *myopia*	3	22.89	0.63	23.17	22.17–23.34	3	24.20	0.29	24.04	24.02–24.53
Female	0	–	–	–	–	0	–	–	–	–
Male	3	22.89	0.63	23.17	22.17–23.34	3	24.20	0.29	24.04	24.02–24.53
CR (mm)										
All	43	7.81	0.24	7.81	7.42–8.27	49	7.76	0.27	7.76	7.21–8.39
*Significant hyperopia*	9	7.82	0.23	7.86	7.59–8.15	6	7.77	0.23	7.79	7.40–8.11
Female	5	7.93	0.22	7.93	7.60–8.15	3	7.63	0.22	7.67	7.40–7.83
Male	4	7.67	0.14	7.62	7.59–7.87	3	7.91	0.18	7.86	7.76–8.11
*Mild* *hyperopia*	31	7.84	0.24	7.81	7.42–8.27	40	7.75	0.28	7.77	7.21–8.39
Female	14	7.81	0.21	7.82	7.42–8.22	22	7.63	0.25	7.69	7.21–8.19
Male	17	7.87	0.27	7.81	7.42–8.27	18	7.90	0.24	7.95	7.35–8.39
*Myopia/risk* *of* *myopia*	3	7.50	0.08	7.47	7.43–7.59	3	7.81	0.28	7.68	7.63–8.13
Female	0	–	–	–	–	0	–	–	–	–
Male	3	7.5	0.08	7.47	7.43–7.59	3	7.81	0.28	7.68	7.63–8.13
SFChT (µm)										
All	36	317	78	318	162–476	48	332	95	321	151–571
*Significant hyperopia*	7	336	88	325	202–454	6	389	141	379	191–571
Female	4	310	100	297	202–443	3	362	193	325	191–571
Male	3	372	71	338	325–454	3	416	101	432	308–508
*Mild* *hyperopia*	26	307	79	305	162–476	39	330	87	321	151–529
Female	12	312	74	285	226–476	21	343	94	371	151–517
Male	14	304	85	318	162–473	18	315	79	303	191–529
*Myopia/risk* *of* *myopia*	3	349	49	362	295–391	3	253	11	247	245–266
Female	0	–	–	–	–	0	–	–	–	–
Male	3	349	49	362	295–391	3	253	11	247	245–266

### Seasonal Variations in Physiological Ocular Growth: Sex and Age Group Differences

It was assumed that those in the *mild hyperopia* group (*n* = 65) would experience physiological ocular growth at this age. A linear mixed-effects model was used to assess seasonal (only the two 5-month intervals) and annual changes in physiological ocular growth in this group, in terms of AL, by sex and age groups and any group interactions—that is, winter–spring (W–S), summer–autumn (S–A2), and annually (A1–A2).

There was a significant decrease in *adjusted* SER annually for 7- to 8-year-old males and females, and over winter–spring for the 7- to 8-year-old males (*F*(12, 183) = 7.64, *P* < 0.001, Holm adjusted *P* ≤ 0.008), but not for those aged 10 to 11 years. There was a significant interaction for season by age group (*F*(3, 189) = 8.22, *P* < 0.001); the 7- to 8-year-olds had a significantly larger decrease in *adjusted* SER over winter–spring and annually than the 10- to 11-year-olds (Holm adjusted *P* ≤ 0.036).

There was a significant elongation in AL over winter–spring, summer–autumn, and annually for each sex in each age group (Fig. 2) (*F*(12, 183) = 70.07, *P* < 0.001, Holm adjusted *P* < 0.018). There was a significant interaction between season and age group (*F*(3, 186) = 30.79, *P* < 0.001); the 7- to 8-year-olds had larger increases than the 10- to 11-year-olds over winter–spring (+0.080 mm) and annually (+0.099 mm, Holm adjusted *P* < 0.001) but not over summer–autumn. The interaction between season and sex was also significant (*F*(3, 186) = 2.89, *P* = 0.037), where males had, overall, larger changes annually than females (+0.034 mm, Holm adjusted *P* = 0.032).

#### Seasonal Variations in Ocular Growth: Differences Between SER Group

To assess differences in ocular growth between SER groups, AL change in the *myopia/risk-of-myopia* group (*n* = 5) and the *significant hyperopia* group (*n* = 14) were compared with those with expected physiological growth (*n* = 65, *mild hyperopia*). A *z*-score with a threshold of ±1.96 (95% confidence level) was used. One child in the *myopia/risk-of-myopia* group (SER: −1.14; *z*-score range: 2.31 to 5.64) and two children in the *significant hyperopia* group (SER range: +2.73 to +5.18; *z*-score range: 2.27 to 3.80) had a larger AL change than those with normal physiological AL change. Three children in the *significant hyperopia* group (SER range: +2.01 to +5.67) had less AL change (*z*-score range: −3.31 to −2.03). For these six children, the AL change exceeded the threshold between one or more of the follow-up periods (winter–spring, summer–autumn, and annually), with no apparent seasonal effect.

#### Monthly Rate of Change in Physiological Ocular Growth per SER Group

**Figure 3.**
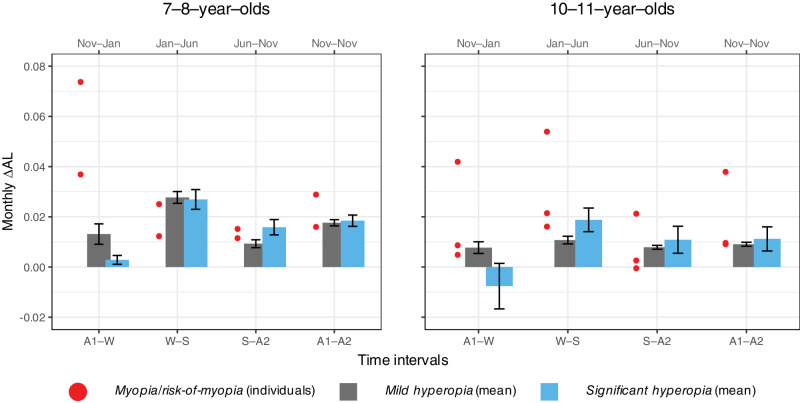
Mean change in AL (∆AL) per month within each season and annually for the 7- to 8-year-olds (*left panel*) and the 10- to 11-year-olds (*right panel*). *Gray* *bars* represent the *mild hyperopia* group (*n* = 30 and 35, *left* and *right panels*), *blue bars* represent the *significant hyperopia* group (*n* = 9 and 5, *left* and *right panels*), and *red dots* represent the individuals in the *myopia/risk-of-myopia* group (*n* = 2 and 3, *left* and *right panels*). *Error bars* represent the mean ± SE.

The monthly rate of change in AL was calculated by dividing ∆AL by the actual number of days within each seasonal-change category (autumn–winter, winter–spring, summer–autumn, and annually) and then multiplying by a nominal 30-day month. For the *mild hyperopia* group, the linear mixed-effects model showed a significant interaction between season and age group (*F*(3, 188) = 6.18, *P* = 0.001); the 7- to 8-year-olds had larger monthly increases than the 10- to 11-year-olds over winter–spring and annually (Holm adjusted *P* < 0.008) but not over autumn–winter or summer–autumn. For both age groups in the *mild hyperopia* group, the highest monthly rate of change was over winter–spring while the lowest was over summer–autumn. For the *significant hyperopia* group and the *myopia/risk-of-myopia group* aged 7 to 8 years, the monthly rate over autumn–winter was the lowest and the highest, respectively (Fig. 3).

### Seasonal Variations in Choroidal Thickness: Differences Between Sex, Age, and SER Groups

#### Differences in ChT Between Sex and Age Groups

**Figure 4.**
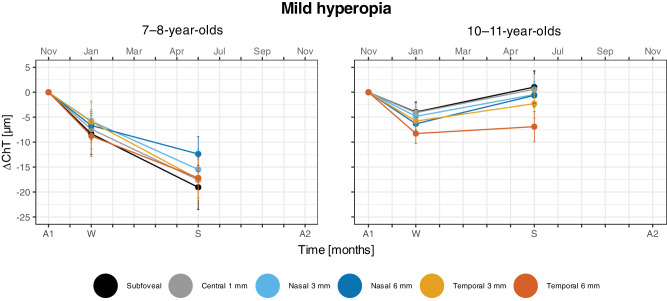
Change in choroidal thickness (∆ChT) by age group across the measurement sessions: autumn 1 (A1), winter (W), and summer (S), normalized by the baseline value from A1. Each area (subfoveal, central 1-mm, nasal and temporal inner [1-mm] and outer [1.5-mm] areas) is indicated by a different color as shown by the legend. The data are for the *mild hyperopia* group (*n* = 25: 7- to 8-year-olds and *n* = 37: 10- to 11-year-olds). *Error bars* represent the mean ± SE.

The linear mixed-effects model showed no seasonal difference in any of the ChT areas (horizontal scans) between sex or age groups. ChT for each group did, however, significantly vary with area (*F*(12, 266) = 2.19, *P* = 0.012, Holm adjusted *P* < 0.012, Supplementary Table S3). The subfoveal, central 1-mm, and temporal inner and outer ChT areas were all significantly thicker than the nasal inner and outer areas for the 7- to 8-year-old females and males and 10- to 11-year-old males. For these three groups, ChT at the nasal inner area was significantly thicker than the nasal outer area. The subfoveal and central 1-mm areas did not differ, nor did either of them differ from the temporal inner and outer areas. Females aged 10 to 11 years differed from these comparisons for the temporal outer area; the subfoveal and central 1-mm areas were significantly thicker than the temporal outer area, and there was no difference between the nasal inner and temporal outer areas. There was a significant interaction between season and age group (*F*(1, 675) = 7.75, *P* = 0.006); the 7- to 8-year-olds had a significantly larger decrease of ChT than the 10- to 11-year-olds over winter–spring (Holm adjusted *P* = 0.006). There was no interaction between season and choroidal area (Fig. 4).

#### Differences in ChT Between SER Groups

To assess differences in ChT between SER groups, comparisons were made between those with expected physiological growth (*n* = 62, *mild hyperopia*), *myopia/risk* *of* *myopia* (*n* = 5), and *significant hyperopia* (*n* = 12) using a *z*-score analysis. Only one child in the *significant hyperopia* group had less change in ChT (central 1-mm, temporal inner, and temporal outer areas) over winter–spring than those with physiological growth (*z*-score range: −2.13 to −1.98). For the remaining 16 children, *z*-score range was −1.81 to 1.59.

#### Associations Between Axial Length and Subfoveal Choroidal Thickness

**Figure 5.**
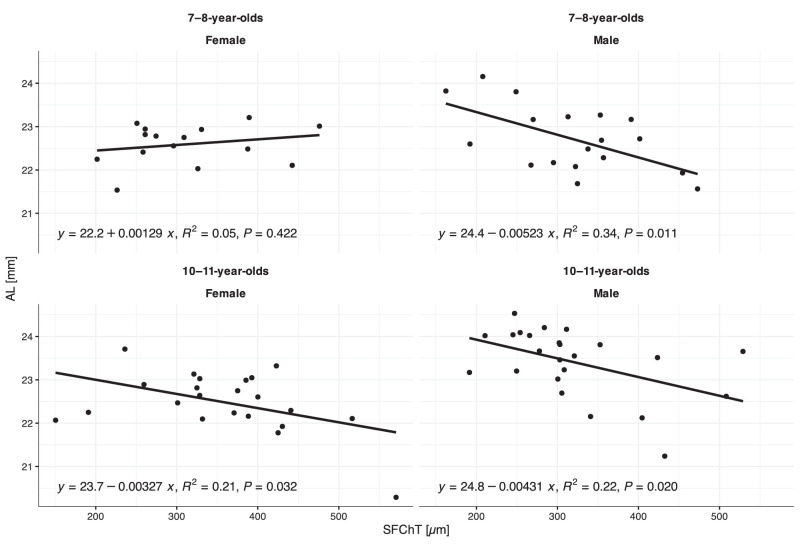
Association between AL and SFChT by sex and age groups in autumn (baseline).

**Figure 6.**
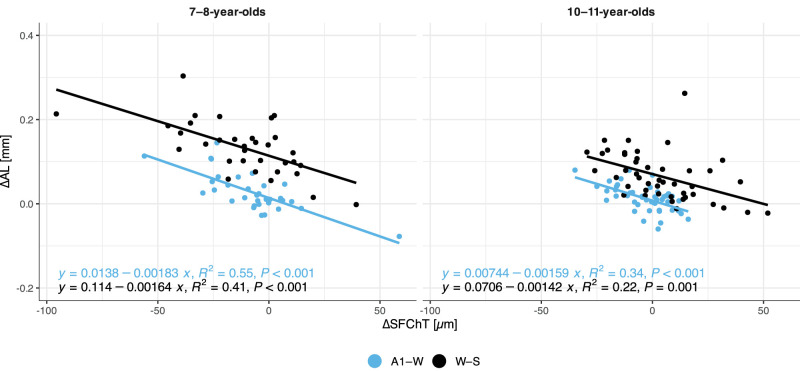
Association between change in axial length (∆AL) and change in subfoveal choroidal thickness (∆SFChT) over autumn–winter (A1 to W) and over winter–spring (W to S). Data from autumn 2 were not included since choroidal thickness was not obtained during that data collection (see Methods section).

Figure 5 illustrates the significant association between longer AL and thinner SFChT, with a significant interaction between SFChT, sex group, and age groups (*n* = 79, adjusted *R*^2^ = 0.40, *P* < 0.001). Figure 6 illustrates the significant association between ∆AL and ∆SFChT, with a significant interaction between age group and season (*n* = 79, adjusted *R*^2^ = 0.65, *P* < 0.001) over autumn–winter and winter–spring for both age groups. There was no association between ∆AL and SFChT over autumn–winter or winter–spring.

### Seasonal Variations in Body Height

The linear mixed-effects model showed a significant increase in body height for both females and males in each age group over all three seasons and annually (*F*(12, 182) = 31.78, *P* < 0.001, Holm adjusted *P* < 0.005), but no interactions between season and sex group or season and age group. There was also no association with change in AL and change in height, for either season or annually.

## Discussion

In this study, we measured ocular parameters in school children aged 7 to 11 years to assess differences across three seasons (autumn–winter, winter–spring, and summer–autumn) and annually, at a latitude (60°N) where there are considerable differences in available daylight over the year. The results show that children experience ocular growth throughout the year, but the rate of growth slows down during the summer–autumn season. This was the case not only in children who had *myopia/risk* *of* *myopia* but also in those who had *mild* and *significant hyperopia*. It was assumed that *mild hyperopes* (cycloplegic SER +0.50 to +2.00 D 7–8 years and +0.25 to +2.00 D 9–10 years)^31^^,^^49^ would experience only physiological ocular growth. The results for this SER group show that there were age and sex differences in physiological ocular growth: (1) the 7- to 8-year-olds had a larger annual AL growth than 10- to 11-year-olds, which was associated with faster AL growth over the winter–spring seasons; (2) males had larger annual AL growth than females, independent of age and season; and (3) AL growth was unrelated to an increase in body height. This coincided with 7- to 8-year-old *mild hyperopes* having a mean annual change in *adjusted* SER of −0.29 D and no associated annual change in SER in the older group. The 7- to 8-year-old *mild hyperopes* also experienced the largest decrease in choroidal thickness over the winter–spring seasons. Similar patterns of change in choroidal thickness were observed in children with *myopia/risk* *of* *myopia* and *significant hyperopia.*

### Seasonal Change in Physiological Ocular Growth

That the rate of physiological ocular growth in mild hyperopes was found to be higher over the winter–spring period compared with summer–autumn resembled that reported for myopic children.^12^^–^^16^ Slowed growth rate over the summer has been associated with an increase in available daylight hours,^15^^,^^16^ which can be paralleled with the reported protective effects of outdoor time against myopia development.^1^^,^^3^^–^^5^ The protective effects of daylight have been hypothesized to be related to its different characteristics (e.g., intensity, spectral composition) compared with indoor electric light. Indeed, exposure to high-intensity illumination has been shown to be critical for optimal refractive development in rhesus monkeys.^6^ What is noteworthy here is that children experienced more physiological growth during the winter—a period when they continue to experience a minimum of 45 minutes of outdoor time during the school day, and this amount of additional daylight exposure has been reported to have a protective effect against myopia.^4^^,^^5^ Though the children in our study had 4 days more at school per month in winter (Table 1), at this latitude, the solar elevation angle (α) is 0° < α < 20° between 06:30 and 16:00 from November to the end of February.^52^ Published data on the spectral composition of daylight from Helsinki, which is at the same latitude as Kongsberg, show that in winter, daylight is both of lower intensity and the spectral composition is blue skewed (blue/green and blue/red ratios >1).^53^ From May to the end of August, the daylight intensity is much higher and the spectral composition is balanced over the same time of day when α > 20° (06:30–16:00) and of lower intensity and becoming blue skewed in the evening when 0° < α < 20°.^52^^,^^53^ Exposure to high-intensity polychromatic daylight, and in particular the short-wavelength part of the spectrum, activates intrinsically photosensitive retinal ganglion cells,^54^^–^^57^ positively affecting diurnal rhythms and dopamine release (if exposure is during morning and day).^55^^,^^58^^,^^59^ Normal melanopsin signaling through modulation of dopaminergic activity plays important roles for the development of the retinal clock network in mice^60^ and, when disturbed, linked with myopia.^61^ In combination with differences in daylight intensity and spectral composition, the children may also spend more time outdoors on nonschool days in summer. As actual outdoor time was not measured in this study, it was not possible to assess to what degree 4 more nonschool days per month (averaged over the summer, Table 1) may also have contributed to the slowed eye growth observed in summer.

### The Relationship Between Choroidal Thickness and Axial Length

In agreement with previous reports, the different areas of the choroid varied in thickness and were overall similar to that reported earlier in children.^62^ The thickness asymmetries in nasal and temporal choroidal areas, in which thinner temporal areas were associated with more physiological ocular growth (Fig. 4, Supplementary Table S3), is the same as that reported for Chinese myopic 12- to 13-year-olds by Tian et al.^63^ They did not find this association for the non-myopes, but the range of refractive errors included in their cohort is not given, so it is difficult to relate directly to our data on mild hyperopes. Furthermore, significant inverse associations were observed between baseline AL and SFChT (Fig. 5) and between ∆AL and ∆SFChT, with the strongest association for the youngest children (Fig. 6).^24^^–^^26^ The results concur with an association between the choroid and physiological ocular axial elongation, both during emmetropization and for maintaining emmetropia. In the younger group, the choroid continues to thin in parallel with continuous physiological axial elongation. In the older group, the choroid is thicker and physiological axial elongation has slowed down. The choroid appears to undergo a thickening process well into adolescence.^62^^,^^64^ Here, the SFChT was on average 23 µm thicker in the 10- to 11-year-old mild hyperopes (Table 3), with minimal changes over the winter (Table 4). The difference between the younger and older group amounts to an increase in SFChT of 7 to 9 µm/year, which is comparable to that reported in other studies.^24^^,^^25^ We surmise that the observed choroidal thickening in 10- to 11-year-old mild hyperopic eyes is a signature of continued, but slowed, physiological ocular growth for maintaining the refractive error.^65^ Choroidal thickening is associated with an increase in choroidal blood flow and increased levels of oxygen and nutrient supply that alter scleral remodeling and growth.^27^ Indeed, it has been shown in animal models of myopia that increased choroidal oxygen supply inhibits the hypoxia inducible factor 1α signaling pathway^66^ that is activated during accelerated ocular growth.^67^ Thus, if myopia onset is a failure to maintain emmetropia/mild hyperopia, then this may be a failure in coordination between the choroid's developmental process, whereby thinning stimulates and thickening inhibits the choroid's response to visual experience that might stimulate ocular growth and development of the ocular components of the eye required for a lasting emmetropic eye.^65^^,^^68^

**Table 4.** Seasonal and Annual Changes in AL, *Adjusted* SER, and Choroidal Thickness by Age Group

		7- to 8-Year-Olds					10- to 11-Year-Olds			
Characteristic	*n*	Mean	SD	Median	Range	*n*	Mean	SD	Median	Range
∆AL (mm)										
*Significant hyperopia*										
A1–W	9	0.006	0.010	0.005	−0.013 to 0.024	5	−0.015	0.039	−0.016	−0.06 to 0.039
W–S	9	0.133	0.060	0.153	0.015 to 0.192	5	0.094	0.053	0.121	0.032 to 0.151
S–A2	9	0.080	0.046	0.082	−0.019 to 0.128	5	0.055	0.061	0.060	−0.021 to 0.129
Annual	9	0.219	0.080	0.260	0.101 to 0.312	5	0.134	0.129	0.086	0.002 to 0.286
*Mild* *hyperopia*										
A1–W	30	0.027	0.042	0.025	−0.077 to 0.113	35	0.015	0.026	0.018	−0.037 to 0.08
W–S	30	0.135	0.066	0.137	−0.002 to 0.304	35	0.054	0.046	0.052	−0.022 to 0.146
S–A2	30	0.047	0.043	0.041	−0.054 to 0.148	35	0.040	0.023	0.037	−0.011 to 0.088
Annual	30	0.209	0.080	0.200	0.106 to 0.505	35	0.108	0.056	0.104	0.015 to 0.231
*Myopia/risk* *of* *myopia*										
A1–W	2	0.109	0.051	0.109	0.073 to 0.145	3	0.035	0.039	0.016	0.009 to 0.08
W–S	2	0.090	0.044	0.090	0.059 to 0.121	3	0.149	0.099	0.107	0.078 to 0.262
S–A2	2	0.069	0.012	0.069	0.06 to 0.077	3	0.040	0.060	0.013	−0.002 to 0.109
Annual	2	0.267	0.108	0.267	0.191 to 0.343	3	0.224	0.196	0.114	0.108 to 0.451
∆*Adjusted* SER (D)										
*Significant hyperopia*										
A1–W	9	−0.03	0.39	−0.02	−0.86 to 0.42	5	0.01	0.11	0.04	−0.15 to 0.13
W–S	9	−0.08	0.25	−0.09	−0.39 to 0.46	5	−0.05	0.29	0.00	−0.45 to 0.31
S–A2	9	−0.11	0.35	−0.15	−0.86 to 0.41	5	0.44	1.31	0.06	−0.43 to 2.73
Annual	9	−0.22	0.27	−0.25	−0.55 to 0.19	5	0.40	1.37	−0.19	−0.35 to 2.85
*Mild* *hyperopia*										
A1–W	30	−0.04	0.27	−0.03	−0.53 to 0.55	35	0.01	0.19	0.04	−0.34 to 0.35
W–S	30	−0.16	0.23	−0.17	−0.71 to 0.58	35	−0.04	0.17	−0.05	−0.31 to 0.32
S–A2	30	−0.09	0.24	−0.04	−0.87 to 0.26	35	−0.04	0.15	−0.03	−0.37 to 0.31
Annual	30	−0.29	0.22	−0.26	−0.82 to 0.07	35	−0.07	0.18	−0.10	−0.46 to 0.37
*Myopia/risk* *of* *myopia*										
A1–W	2	−0.21	0.12	−0.21	−0.3 to −0.12	3	0.07	0.16	0.04	−0.07 to 0.25
W–S	2	0.06	0.48	0.06	−0.28 to 0.4	3	−0.36	0.13	−0.37	−0.48 to −0.22
S–A2	2	−0.23	0.02	−0.23	−0.25 to −0.22	3	−0.08	0.17	−0.10	−0.24 to 0.09
Annual	2	−0.38	0.62	−0.38	−0.82 to 0.06	3	−0.37	0.38	−0.24	−0.8 to −0.07
∆SFChT (µm)										
*Significant hyperopia*										
A1–W	6	−1	9	0	−17 to 10	6	1	3	1	−4 to 4
W–S	6	−18	20	−17	−45 to 4	6	−5	17	−7	−29 to 18
*Mild* *hyperopia*										
A1–W	25	−8	21	−7	−56 to 59	37	−4	12	−1	−35 to 16
W–S	25	−11	27	−6	−96 to 39	37	5	19	3	−26 to 52
*Myopia/risk* *of* *myopia*										
A1–W	2	−15	11	−15	−23 to −7	3	−4	6	−3	−10 to 1
W–S	2	−4	21	−4	−18 to 11	3	11	17	15	−7 to 26

Refractive error grouping is the same as in Table 3. Only children who completed all four measurements were included for AL (*n* = 84) and *adjusted* SER (*n* = 84) and three measurements for SFChT (*n* = 79).

### Annual Changes in Physiological Ocular Growth and Spherical Equivalent Refractive Error

The annual change in AL for those undergoing physiological ocular growth was 0.21 mm and 0.11 mm for 7- to 8-year-olds and 10- to 11-year-olds, respectively (*mild hyperopia* group in Table 4). This is comparable to the average annual axial elongation reported for emmetropic children from age 6 to 9 years in the Netherlands (0.19 mm/year).^69^ Corneal radii appear to change very little after early childhood,^68^^–^^71^ which was similar to our results (average annual change of 0.007 mm, results not shown). The larger annual AL change in the 7- to 8-year-old children corresponded with a significant annual decrease in *adjusted* SER (Table 4). That the annual rate of physiological ocular growth has been reported to slow down from after the age of 9 (cf. Table 4 and Figure 3D in Zadnik et al.^29^) is corroborated by our results for 10- to 11-year-olds. The continued, but slowed, AL growth without an associated change in SER for the older children indicates that they have entered a phase whereby they maintain their emmetropia/mild refractive error (through a coordinated decrease in crystalline lens power^30^^,^^72^). When active emmetropization completes and transitions to maintenance of a mild refractive error, however, varies between individuals. First, we observed that the 7- to 8-year-old males had a larger decrease in SER (but same increase in AL) than their female peers. Thus, that females appear to exhibit faster myopic progression at an earlier age (measured by SER) than their male peers^73^^,^^74^ could be due to emmetropization completing, on average, at an earlier age for females. Second, albeit with a small sample (Table 4 and Fig. 3), some significant hyperopes have a continued decrease in SER and more-than-physiological AL growth at ages 10 to 11 (but their growth pattern appears to be different from that of emmetropes and myopes^28^). Both instances were associated with thinning of the choroid.

Physiological ocular growth is desirable as part of emmetropization (for a review, see Flitcroft^28^) and as part of coordinated growth for maintaining a mild refractive error throughout adolescence.^30^ The bulk of this growth happens in winter, and it seems that for emmetropization to complete around mild hyperopia,^31^ slowing of growth is needed in summer. To maintain physiological rather than accelerated growth from winter to spring, the slowing of ocular growth needs to be accompanied by development of a thicker and more resilient choroid^27^^,^^30^^,^^72^^–^^74^ over the summer–autumn. This resilience appears to decay, as the monthly AL increase was slower over autumn–winter compared with winter–spring (Fig. 3).

### Sex Differences in Ocular Biometry

At baseline (Table 3), there were no differences in AL between males and females aged 7 to 8 years, but the 10- to 11-year-old males had almost 1-mm longer eyes than peer females. Males also had significantly flatter corneal radii than females (0.06–0.27 mm), resulting in no differences in SER between sexes. In the Generation R study,^69^ males had a significantly longer eye and flatter corneas than females at both 6 years (0.5 and 0.14 mm) and at 9 years (0.52 and 0.13 mm), but their sample included also hyperopes and myopes (cf. their Table 2).

There were no seasonal differences in AL elongation between the sexes, but as observed in a study including children aged 10 to 15,^32^ males had a small but significantly larger annual change in physiological ocular growth than females (0.034 mm), independent of age.

### Body Height

In line with previous reports,^75^^–^^77^ a significant but weak association between baseline body height and AL was observed. The annual increase in body height was as expected from reported growth curves for Norwegian 7- to 11-year-old children.^78^ There was no association between ∆AL and ∆body height as reported in another study on primary school children.^79^

### Strengths and Limitations

The study benefited from the cohort having mandatory outdoor time during recess every school day irrespective of season. Though daylight exposure was not measured, all would have had a minimum of 45 minutes of daylight exposure every school day. Thus, when we use recess time as a proxy, all would have exceeded the 40 minutes of outdoor time per day reported to decrease myopia incidence.^5^ Considering additional outdoor time when walking/cycling to and from school and some outdoor time during the BAS program, it is not unreasonable to assume that most children would have had 1 to 2 hours of outdoor daylight exposure every school day throughout the year. It is a limitation that we did not obtain objective measurements of personal light exposure. This would be needed to quantify differences in (1) dose–response (intensity × duration) variation in winter versus summer and (2) exposure to the shorter wavelengths of the spectrum in the evening over spring–summer, like that reported for adults.^80^

Another strength is that each child was measured over a 30-minute period within a 3.5-hour window around midday to account for any diurnal variation. However, prior to measurement, children went about their school day as normal, including outdoor recess. Since, at that time of the day, any child would not have been outdoors for more than 30 minutes and would have spent up to 30 minutes in the measurement room with light levels below 110 lux during the 15 minutes prior to OCT imaging, choroidal thinning as a result of short-term outdoor time should have been neutralized.^23^

The study was limited by only having a single cycloplegic autorefraction measurement. Taking cycloplegic measurements at all time points was considered too disruptive to the children's school day and would have been impractical during COVID-19. Crystalline lens power and refractive errors with cycloplegia are important when assessing changes during ocular development, as uncontrolled accommodation can contribute to measuring more negative values of SER.^81^^–^^83^ To circumvent this, we modeled *adjusted SER* based on the measured cycloplegic SER with AL/CR, non-cycloplegic SER, and age as predictors. This resulted in reasonable estimates of changes in SER.^68^

Another limitation is the small number of children with *myopia/risk* *of* *myopia* and *significant hyperopia.* The frequencies of refractive errors, however, are in line with that reported for children and adolescents in this region of the world.^7^^–^^9^ Additionally, that 3 of the 6 myopes (−0.50 to −1.25 D) and 11 of the 15 significant hyperopes (+2.00 to +3.00D) were uncorrected during the parts of the study limits the generalizability of the results for these refractive error groups. Previous studies have reported that both accommodation^84^ and defocus^41^ can influence choroidal thickness, potentially affecting the uncorrected hyperopes and the uncorrected myopes, respectively.

It is unlikely that the short COVID-19 lockdown with homeschooling that included outdoor recess, when compared with lockdowns in other countries,^85^ would have affected the measurements in June 2020 and November 2020. Thus, observed changes of the mild hyperopes appear to be related to physiological ocular growth. COVID-19 restrictions did prevent the collection of OCT measurements at the last time point (November 2020), preventing assessment of changes in ChT over the summer–autumn season and annually.

Lastly, we used Zadnik's age-sensitive cutoff points for SER^49^ to assess physiological ocular growth (*mild hyperopes*, assuming that this is a more natural endpoint for emmetropization)^31^ and to identify children with myopia risk. This resulted in a higher threshold for SER than typically used for emmetropia (−0.50 < SER <+0.50), reducing the likelihood that those assumed to experience physiological ocular growth would have been pre-myopic at baseline.^86^

## Conclusions

There were significant seasonal and annual changes in AL in children irrespective of refractive error, notably in children assumed to experience only normal physiological eye growth irrespective of age. The results confirm that the time of year and the frequency at which children have eye examinations are important factors when assessing myopia risk and scheduling of any needed myopia control treatment.^15^ Annual changes in AL were smaller and the choroid was thicker in 10- to 11-year-old *mild hyperopes*. Annual decline in SER and seasonal ChT thinning were observed in 7- to 8-year-old but not 10- to 11-year-old *mild hyperopes*, supporting the notion that the 7- to 8-year-olds are still undergoing emmetropization, while the 10- to 11-year-olds have transitioned to maintaining emmetropia. That mild hyperopes have more ocular growth over winter suggests that human physiological ocular growth may follow a seasonal cycle linked with the availability and variability in intensity and spectral composition of daylight.

## Supplementary Material

Supplement 1
