# Supplementary material for: Seasonal and Annual Change in Physiological Ocular Growth of 7- to 11-Year-Old Norwegian Children
Source: Invest Ophthalmol Vis Sci. 2023 Dec 8;64(15):10. doi: 10.1167/iovs.64.15.10 (PMC10709800; doi:10.1167/iovs.64.15.10)
Supplement: Supplement 1 [file iovs-64-15-10_s001.docx]

Supplementary material

Seasonal and annual change in physiological ocular growth of 7–11-year-old Norwegian children

Nickolai G. Nilsen^1^

Stuart J. Gilson^1^

Helene Lindgren^1^

Marianne Kjærland^1^

Hilde R. Pedersen^1^

Rigmor C. Baraas^1^

^1^ National Centre for Optics, Vision and Eye Care, Faculty of Health and Social Sciences, University of South-Eastern Norway, Kongsberg, Norway

**Table S1.** Within-session SD, 95% confidence interval (CI), median and 1^st^ and 3^rd^ quartile, and difference for the individual with the maximum value for measurements of cycloplegic SER, non-cycloplegic SER, and axial length. Within-session SD was obtained from a linear mixed effects model. CI was obtained by profiling the likelihood. Median and interquartile range were estimated from each individual using *t*-statistics.

|  | **n** | **Within-session SD**  (95% CI) | **Median**  (interquartile range) | **Largest difference** |
| --- | --- | --- | --- | --- |
| Cycloplegic SER (D)  (HRK-8000A) | 78 | 0.07 (0.065–0.073) | 0.05 (0.03–0.08) | ±0.32 |
| Non-cycloplegic SER (D)  (Nvision-K 5001) | 81 | 0.21 (0.19–0.23) | 0.18 (0.13–0.30) | ±0.70 |
| Axial length (mm)  (IOLMaster 700) | 83 | 0.005 (0.005–0.005) | 0.004 (0.003–0.005) | ±0.015 |

**Model comparisons for an adjustment of non-cycloplegic SER**

To limit the underestimation of hyperopia and overestimation of myopia when classifying refractive error, an *adjustment* of non-cycloplegic SER measurements was needed for the 13 participants for whom cycloplegic SER was not obtained in January (W), and for all participants in A1, S and A2.

A quantile regression^1^(Eq. 1) was calculated using cycloplegic SER as an independent variable, and the combination of AL/CR ($x_{1}$), non-cycloplegic SER ($x_{2}$) and age ($x_{3}$) as predictors, resulted in the most optimal model (M3, Supplementary Table S1). Model M3 had the least misclassifications in grouping of participants compared to cycloplegic SER, and Bland-Altman analysis^2^ when comparing non-cycloplegic (Nvision-K 5001) and cycloplegic SER (HRK-8000A), and then adjusted SER and cycloplegic SER show that the mean difference was reduced from -0.83D to -0.06D between the two refraction measures, respectively (Supplementary Figure S1).

$Q_{\tau}(y_{i})=\beta_{0}\left( \tau\right)+\beta_{1}\left( \tau\right)x_{1,i}+\beta_{2}{\left( \tau\right)x}_{2,i}+\beta_{3}{\left( \tau\right)x}_{3,i}$ (Eq. 1)

where $i$ is the $i$-th participant, $\tau$ = 0.5 is the median quantile, and $\beta_{0\ldots3}$are the coefficients. The coefficients obtained from equation (Eq. 1) were used to predict the *adjusted* SER for classification and grouping of participants according to refractive error.

**Table S2.** Comparisons of quantile regression models using different predictors. Goodness of fit for comparing models was estimated as described by Koenker and Jose ^3^. Cycloplegic SER median (range) was +1.05 (-1.14–+5.67).

|  | **Predictors** | **Goodness of fit** | **Median (range)** | **# Misclassification compared with cycloplegic SER** | **Accuracy in grouping compared to cycloplegic SER** |
| --- | --- | --- | --- | --- | --- |
| M1 | AL/CR | 0.31 | +1.23 (-0.37–+2.63) | 15 | 0.81% |
| M2 | AL/CR, non-cycloplegic SER | 0.40 | +1.15 (–1.14–+4.48) | 15 | 0.81% |
| M3 | AL/CR, non-cycloplegic SER, age | 0.41 | +1.19 (-1.00–+4.12) | 12 | 0.85% |

**Figure S1.** Bland-Altman plots (n=78): (A) Non-cycloplegic SER versus cycloplegic SER. Mean difference was -0.83, 95% confidence interval = -2.24–0.58. (B) *Adjusted* SER (from quantile regression equation using AL/CR, non-cycloplegic SER and age) versus cycloplegic SER. Mean difference was -0.06, 95% confidence interval = -1.27–1.15.

**Table S3.** Multiple comparisons between choroidal thicknesses at the different areas of the choroid. Results from linear mixed effects model. P-value is Holm-adjusted for multiple comparisons (p<0.01**, p<0.001***)

|  | 7–8-year-olds | | 10–11-year-olds | |
| --- | --- | --- | --- | --- |
| **Comparison** | **Females** | **Males** | **Females** | **Males** |
| SFChT vs. central 1 mm | p=1.0 | p=1.0 | p=1.0 | p=1.0 |
| SFChT vs. nasal inner | *** | *** | *** | *** |
| SFChT vs. nasal outer | *** | *** | *** | *** |
| SFChT vs. temporal inner | p=1.0 | p=0.7 | p=1.0 | p=1.0 |
| SFChT vs. temporal outer | p=1.0 | p=0.5 | *** | p=0.6 |
| Central 1 mm vs. nasal inner | *** | *** | *** | *** |
| Central 1 mm vs. nasal outer | *** | *** | *** | *** |
| Central 1 mm vs. temporal inner | p=0.9 | p=1.0 | p=1.0 | p=1.0 |
| Central 1 mm vs. temporal outer | p=1.0 | p=1.0 | *** | p=0.7 |
| Nasal inner vs. nasal outer | *** | *** | *** | *** |
| Nasal inner vs. temporal inner | *** | *** | *** | *** |
| Nasal inner vs. temporal outer | ** | *** | p=1.0 | ** |
| Nasal outer vs. temporal inner | *** | *** | *** | *** |
| Nasal outer vs. temporal outer | *** | *** | *** | *** |

**References**

1. Hao L, Naiman DQ. *Quantile Regression*: SAGE Publications; 2007.

2. Bland JM, Altman DG. Statistical methods for assessing agreement between two methods of clinical measurement. *International Journal of Nursing Studies* 2010;47:931-936.

3. Koenker R, Jose AFM. Goodness of Fit and Related Inference Processes for Quantile Regression. *Journal of the American Statistical Association* 1999;94:1296-1310.
